# Yoghurt-Type Gels from Skim Sheep Milk Base Enriched with Whey Protein Concentrate Hydrolysates and Processed by Heating or High Hydrostatic Pressure

**DOI:** 10.3390/foods8080342

**Published:** 2019-08-12

**Authors:** Lambros Sakkas, Maria Tzevdou, Evangelia Zoidou, Evangelia Gkotzia, Anastasis Karvounis, Antonia Samara, Petros Taoukis, Golfo Moatsou

**Affiliations:** 1 Laboratory of Dairy Research, Department of Food Science and Human Nutrition, Agricultural University of Athens, Iera Odos 75, 118 55 Athens, Greece; 2 Laboratory of Food Chemistry and Technology, School of Chemical Engineering, National Technical University of Athens (NTUA), Laboratory of Food Chemistry and Technology, 5 Heroon Polytechniou Str., Zografos, 15780 Athens, Greece

**Keywords:** high hydrostatic pressure, whey protein hydrolysates, sheep milk, yoghurt, ACE inhibitory activity, gel properties, heat stability, traditional yoghurt starter, biofunctionality

## Abstract

An objective of the present study was the enrichment of skim sheep yoghurt milk base with hydrolysates (WPHs) of whey protein concentrate (WP80) derived from Feta cheesemaking. Moreover, the use of high hydrostatic pressure (HP) treatment at 600 MPa/55 °C/10 min as an alternative for heat treatment of milk bases, was studied. In brief, lyophilized trypsin and protamex hydrolysates of WP80 produced under laboratory conditions were added in skim sheep milk. The composition and heat treatment conditions were set after the assessment of the heat stability of various mixtures; trisodium citrate was used as a chelating agent, when needed. According to the results, the conditions of heat treatment were more important for the physical properties of the gel than the type of enrichment. High pressure treatment resulted in inferior gel properties, irrespective of the type of enrichment. Supplementation of skim sheep milk with whey protein hydrolysates at >0.5% had a detrimental effect on gel properties. Finally, skim sheep milk base inoculated with fresh traditional yoghurt, resulted in yoghurt-type gels with high counts of *Lb. delbrueckii* subsp. *bulgaricus* and *Str. thermophilus* -close to the ideal 1:1- and with a high ACE inhibitory activity >65% that were not essentially affected by the experimental factors.

## 1. Introduction

The manufacture of low-fat, high-protein fermented milks is one of the current trends in food technology. High-protein, high-mineral set-style yoghurt manufactured from partially defatted or skim sheep milk can be stable throughout four weeks of storage. However, the fat removal reduces the water holding capacity and the firmness of the skim yoghurt in comparison to the reduced-fat counterpart [1]. An objective of the present study was the enrichment of the skim sheep milk base with hydrolysates (WPHs) of whey protein concentrate (WP80) derived from Feta cheesemaking. The hypothesis was that this intervention could modify the texture and increase the biofunctionality, in terms of angiotensin converting enzyme inhibitory activity (ACE-IA), in accordance to our recent findings for reduced-fat cow milk yoghurt [2]. Fortification or enrichment of yoghurt cow milk base with WPC has been widely studied [3,4], but similar interventions in small ruminants yoghurt milk are scarce and have been applied in goat milk [5,6,7].

Hydrolysis of whey or individual whey proteins or whey protein concentrates by various enzymes results in mixtures of proteins and peptides known as whey protein hydrolysates (WPHs). WPHs can exhibit improved biofunctionality and modified physical properties compared to the substrate [4,8,9]. The use of whey protein hydrolysates (WPHs) in the cow milk yoghurt base has been studied in regard to the growth of probiotics [10,11] or biofunctional and textural properties [2,12].

An important and essential step of the manufacture of fermented milks is the heat treatment of the milk base. The heat stability of sheep milk is lower than cow milk due to differences in the casein micelle structure and mineral content [13,14]. Therefore, another objective of the present study was the use of high hydrostatic pressure (HP) as an alternative for the heat treatment of yoghurt milk bases. HP processing has been associated with variable effects on yoghurt properties that depend on the composition of cow milk base [15,16,17]. However, information for the effect of HP-treatment on sheep milk that could affect yoghurt manufacture and properties is limited [18,19,20,21,22,23]. 

## 2. Materials and Methods

### 2.1. Hydrolysis of Whey Protein Concentrate 

The substrate was a 5% (*w*/*w*) aqueous dilution of whey protein concentrate powder with approximately 80% (*w*/*w*) protein content (WP80) derived from sheep/goat Feta cheese whey (Epirus Protein SA, Ioannina, Greece). The composition of WP80 (Table 1) was determined as described by Zoidou et al. [7]. The aqueous dilution of WP80 remained overnight at 4 °C–6 °C for proper hydration of the powder. Hydrolysis was carried out by means of trypsin and Protamex. The ratio enzyme to protein was 0.25%, and 0.1% for trypsin and protamex, respectively. Incubations were carried out at 50 °C without pH adjustment at pH 6.2–6.3, which was the pH of the WP80 dilution. After 60 min, the pH was 5.6–6.1 and hydrolysis was stopped by heat treatment at 68 °C for 10 min, which are the conditions for batch pasteurization of milk. Hydrolysates were powdered by lyophilization. Trypsin and protamex hydrolysates were symbolized as WPHt and WPHp. Hydrolysis conditions resulted from previous experiments (data not shown).

### 2.2. Assessment of Heat Stability of Various Sheep Milk Bases 

The aim of this experimental section was the selection of heat stable milk bases that could be used for the manufacture of yoghurt-type gels. Raw sheep milk was defatted by centrifugation at 3000× *g* for 20 min at 40 °C. Skim sheep milk (0.1% fat) was mixed with variable quantities (0.5%–2% *w*/*w*) of WP80, WPHt and WPHp powders with or without trisodium citrate at 0.2% (*w*/*w*), i.e., approximately 7 mM. Experiments were performed in duplicate and three replicates of each mixture were prepared in each experiment. Mixtures were kept overnight at 5 °C–6 °C for hydration. Then, two of the replicates were heated at 70 °C, 75 °C, 80 °C, 85 °C, 90 °C and 95 °C for 5 min. At the end of the heat treatment, the milk mixtures were cooled down immediately at room temperature using an ice-bath. One of the triplicates was not heated (control). Heat-treated (HT) and control mixtures were centrifuged at 10,000× *g* for 10 min. Appearance of sediment indicated heat instability. 

### 2.3. Heat- and High Pressure- Treatment of Selected Skim Sheep Milk Bases

Raw ovine milk was defatted by means of a lab-scale milk fat separator. The composition and treatments of milk bases used in the subsequent yoghurt-type gel experiments are shown in Table 2. The criterion for their selection was the heat stability (Section 2.2). Heat treatments (HT) were carried out under batch conditions. The same mixtures without trisodium citrate were processed by high pressure (HP) as follows. Treatments were performed using a laboratory-scale HP system with a maximum operating pressure of 1000 MPa (Food Pressure Unit FPU 1.01, Resato International BV, Roden, Netherlands), consisting of an HP unit with a pressure intensifier, an HP vessel of 1.5 L and a multi-vessel system consisting of six vessels of 42 mL capacity each. All HP vessels are surrounded by a water circulating jacket connected to a temperature control system. The pressure-transmitting fluid used was the polyglycol ISO viscosity class VC 15 (Resato International BV, Netherlands). Milk bases were put into a multilayer (PP, foil, PE) packaging and placed in the 1.5 L chamber for processing. The desired value of pressure was set and, after pressure build-up (ca. 20 MPa·s-1), the pressure vessel was isolated; this point defined the zero time of the process. The pressure of the vessel was released after a preset time interval (10 min pressurization time) by opening the pressure valve (release time <3 s). The initial temperature increase during the pressure build-up (ca. 3 °C per 100 MPa) was taken into consideration in order to achieve the desired operating temperature. The pressure and temperature were constantly monitored (intervals of 1 s) and recorded during the process. All samples were pressurized at 619 ± 7 MPa and 55.2 ± 1.3 °C for a process time of 10 min. After processing, samples were kept overnight at 4 °C. The heat and high-pressure experiments were performed in duplicate.

### 2.4. Manufacture of Yoghurt-type Gels

After treatments, the temperature of the mixtures of Table 2 was adjusted to 43 °C, which was the inoculation temperature. Fresh Greek traditional sheep yoghurt with the characteristic top fat layer [24] was used as a starter, at a ratio of 1.5% (*v*/*v*). Portions of 100 mL of inoculated milk bases were poured in sterilized containers, in which they were incubated at 42 °C, until pH 4.7. Incubation was stopped by immediate cooling. At the end of cooling, the yoghurt-type gels had pH 4.6. The average duration of incubation was 2 h and 30 min and 2 h and 18 min for HT and HP treated mixtures, respectively. 

### 2.5. Analyses

All analyses were performed as previously described [1,2,7]. In brief, gross composition, yoghurt bacteria counts, water holding capacity (WHC), firmness/cohesiveness and ACE-IA determinations were carried out by means of the Milkoscan, colony count technique, centrifugation, texture profile analysis and RP-HPLC, respectively. 

### 2.6. Statistical Analysis

The analysis of variance (ANOVA) was used to analyze the effect of the mixture composition (milk base) and of the treatment on the characteristics of the yoghurt-type gels and for the differences among the means of the Least Significant Difference test was used (LSD, *p* < 0.05). Statistical analysis was carried out by means of the Statgraphics, Centurion V (Manugistics Inc., Rockville, MA 20852, USA).

## 3. Results and Discussion

Sodini et al. [3] summarized that the rheological properties and microstructure of yoghurt are related to the heating conditions of the milk base from 75 °C for 1 min–5 min to 95 °C for 5 min–10 min; the later conditions result in >99% denaturation of the β-lactoglobulin (β-lg). The heat treatment induces the formation of various types of complexes, which are key factors for the configuration of acid milk gels and yoghurt: i. between casein micelles and denatured whey proteins, ii. between κ-casein and β-lg and iii. between denatured whey proteins [25]. Their extent and distribution are differentiated within the range from pH 6.5 to pH 6.7. At low pH, the denatured whey proteins bind onto the casein micelles thus increasing the particle size while at higher pH they participate in soluble complexes with solubilized κ-casein [26]. 

From Table 2, it is evident that only the control skim sheep milk base was stable under the usual heating conditions for the yoghurt manufacture –i.e., 95 °C/5 min. The addition of WPC or WPHs even at low level decreased the heat stability and trisodium citrate addition was necessary. The heat stability of sheep milk is lower than that of cow milk because ovine micelles are more mineralized, contain more β-casein, are less hydrated than their bovine counterparts and their size increase substantially during heating. In fact, heating at >80 °C causes an increase of the ovine micelle size by >50%, which along with the high casein content favour micelle-micelle interaction and aggregation [13,14]. The later phenomenon is expected to be more pronounced in the skim sheep milk of the present experiments due to the increase of protein concentration caused by the removal of fat. Moreover, the calcium content of WPC and WPHs may also adversely affect the heat stability of the mixtures of Table 2. Excessive calcium before heat treatment results in the formation of large aggregates that decrease the heat-stability while calcium combined with acidification induce the gelation of denatured whey protein polymers [27].

Therefore, both the low pH and the elevated calcium content of the milk bases before acidification could be responsible for the poor heat stability of the sheep milk bases in the experiments of Section 2.2. Milder heating conditions and the addition of trisodium citrate before heat treatment were used for the enriched skim milk bases. As shown in Table 2, the pH of milk bases supplemented with approximately 7 mM sodium citrate was higher compared to the control before and after heat treatment, on average by 0.12 pH and 0.16 pH units, respectively. The addition of sodium citrate in sheep or goats milk enhances their heat stability by linking with ionic calcium and solubilizing both the colloidal calcium phosphate and the calcium linked to the phosphoseryl residues. Moreover, sodium citrate increases the milk pH by approximately 0.1 units resulting in the increase of small-sized casein micelles and of their negative charge that do not favour heat aggregation [13,14]. In regard to cow milk yoghurt gels, using >25 mM trisodium citrate causes detrimental disruption of casein micelles, while the addition of 10 mM–20 mM trisodium citrate improves the texture by chelating calcium. Calcium chelation induces solublization of colloidal calcium phosphate, thus enhancing the formation of crosslinks [28]. The milk bases were stable under the conditions of HP treatment, which reduced the pH by 0.18 units, on average. 

### 3.1. Properties of Yoghurt-type Gels

The effect of the experimental factors on the physical and compositional properties of yoghurt-type gels was analyzed by the multifactor ANOVA and it is shown in Table 3. 

It is evident (Table 3) that the physical properties (water holding capacity, firmness and cohesiveness) were affected significantly (*p* < 0.05) by the type of enrichment (composition) of the milk base and by the processing (heat- or high pressure-treatment). Moreover, there was a significant (*p* < 0.05) combined effect of these factors (A × B) on the physical properties. The days of storage affected significantly (*p* < 0.05) the pH and the firmness of gels.

The physical and compositional properties of the yoghurt-type gels treated by heat or by high-pressure are presented in Table 4. The factor “composition”- that is the composition of the milk base mixture—was related to statistically significant differences (*p* < 0.05) mostly in the HT group. These differences can be assigned to: i. different heat treatments, and ii. differences in composition. It is evident that at day three, the YWPHt1 base treated at 75 °C for 5 min, i.e., under the mildest conditions, was significantly differentiated among the HT group in regard to the water holding capacity (WHC), firmness and cohesiveness. The extend of whey protein denaturation in YWPHt1 was expected to be the lowest, which is consistent with its lowest WHC. Extended heat denaturation of β-lg enhances the capability of the casein network to immobilize the serum [3]. On the other hand, in high-protein yoghurts the reduction of heating temperature from 95 °C to 75 °C for 5 min reduces firmness but improves the sensory properties [29]. Despite the highest total solids and protein content of YWPHt1, its firmness was the lowest in accordance to the above-mentioned effect of heating on the formation and distribution of complexes. It has to be noticed that the enrichment of skim milk - i.e., higher total solids and protein contents - and heat treatment at 85 °C or 90 °C for 5 min resulted in significantly higher (*p* < 0.05) WHC and firmness for YWP80, YWOHp0.5 and WPHt0.5 compared to the control Y0. Therefore, the supplementation with WP80 at 1% and with WPHs at 0.5% counteracted the effect of milder heating conditions. The addition of whey proteins in the yoghurt milk base increases also the ratio whey protein to casein that in turn increases WHC and affect also the viscoelasticity and flow behavior; the latter is related to heating conditions [3]. In particular, the whey protein to the casein ratio has been demonstrated as a crucial factor for the structure of non-fat stirred yoghurts [30]. The enrichment with WPHs resulted in lower WHC and firmness compared to the intact WP80. Apparently, due to hydrolysis, the WPHt and WPHp contained less intact native whey proteins, which are key components for the crosslinking within the yoghurt gel matrix, as reported above. Moreover, the solubility of a WPH may be reduced due to the exposure of hydrophobic areas of the molecules [9].

Similar findings have been reported by other researchers. The use of the WPHt of the present study in reduced-fat cow milk base did not influence WHC but dramatically affected firmness, while the use of commercial WPHs of bovine origin had the opposite effect [2]. The addition of commercial WPHs in reduced-fat cow milk base at levels lower than 0.4% (*w*/*v*) affected negatively the texture of yoghurts due to less cross-linked microstructure [11]. The use of a tryptic WPH in buffalo milk at a ratio of 3% decreased the firmness and increased the syneresis of the sweetened yoghurt made there from [12].

The observation that the heating conditions were more important than the enrichment of the sheep milk base coincides with the findings for the HP-group of yoghurt-type gels. Less differences were observed between the HP milk bases (Table 2). These differences can be attributed solely to the enrichment since all were treated under the same conditions. Similar HP treatments of full-fat sheep milk induced >90% denaturation of β-lg and substantial reduction of α-la [22]. Firmness of the HP-group was significantly lower compared to the HT-group (Table 4) and this holds true for the control non-supplemented milk base Y0. WHC was not affected but firmness of YWP80 supplemented with 1% WP80 was significantly the lowest, half that of the control Y0. The opposite was true for cohesiveness. From the literature reviews [3,16,17] comes out that according to several studies, the HP treatment improve firmness and WHC. However, there are also opposite reports that coincide with our findings [15,29]. Similarly to the present study, the HP treatment of the cow milk base supplemented with whey protein concentrates and isolates resulted in weaker and less firm acid gels compared to heat treatment due to differences in the complexation of denatured β-lg [31,32]. The study of the effect of HP on reconstituted skim milk powder base combined with heating and in comparison to heat-treatment suggested that it can be used for the production of high protein drinking yoghurts of low viscosity [33]. Interestingly, the incorporation of WPHs in the HP treated milk bases had less detrimental effects on physical properties than the addition of non-hydrolyzed WP80. Again, a possible explanation is a favourable change of solubilization and the interactions between peptides and proteins, induced by partial hydrolysis [9].

The HP treatment of homogenized pasteurized full-fat sheep milk at 500 MPa/55 °C increase the firmness and the WHC of yoghurt compared to the typical 95 °C/5 min heat treatment [19]. The effect of HP on skim sheep milk has not been reported, but it is reasonable to expect that its high protein content could affect its behavior under various HP conditions. Additionally, the particularities of sheep milk and casein should be taken into consideration. Our previous study [23] showed that HP conditions similar to the present study affected the rennet clotting behavior of full-fat sheep milk in a different manner compared to cow milk. HP at 600 MPa decreased the size of ovine micelle by 40% [21]. At 400 MPa the κ-casein was extensively solubilized by >80%, much higher than the 22% observed in ovine milk [18]. Therefore, it could be assumed that soluble κ-casein/β-lg complexes were favoured under the applied HP conditions impairing thus crosslinking between micelles and β-lg and consequently the gel microstructure. 

### 3.2. Biofunctional Characteristics

The thermophilic counts are “components” of yoghurts with biological value and along with particular substances such as proteins, peptides, vitamins, specific lipid compounds configure the biofunctionality of this type of fermented milks. Thermophilic bacilli and cocci log counts—estimated according to the ISO 7889-IDF 117 standard [34] - and the percentage of angiotensin converting enzyme inhibitory activity (ACE-IA)—often called anti-hypertensive potential- are shown in Table 5. 

According to the results, both the enrichment and the treatment of the milk base did not affect the counts of *Lb. bulgaricus* and *Str. thermophilus*, which were high and very close to the suggested 1:1 ratio, after 10 days of storage. Of particular interest are the high numbers of bacilli which are considered very beneficial for the gastrointestinal system. The modern trend for yoghurts and fermented milks with mild characteristics has suppressed the presence and the viability of *Lactobacillus delbrueckii* subsp. *bulgaricus* in yoghurt starters to avoid the development of excessive acidity during storage. In our previous experiments carried out with commercial starters [1,7], we estimated less than four log cfu/g thermophilic bacilli whereas cocci counts were close to nine log cfu/g. Very low or even zero lactobacilli counts have also been estimated in market yoghurts [35]. Apparently, the biofunctional profile of the yoghurt-type gels of Table 5 is related to the Greek traditional sheep yoghurt used as a starter that contained 8.52 log cfu/g *Lb. bulgaricus* and 6.82 log cfu/g *Str. thermophilus*, while its ACE-IA was 57.5%.

ACE-IA in the present gels was high coinciding with the high bacilli counts and the high % ACE-IA of the starter. In our previous studies [1,7] the ACE-IA of the skim sheep milk set-style yoghurt ranged from 22% to 25%. In the present study, ACE-IA was not affected by the type of treatment and statistical differences were observed only between different mixtures of the HT-group. In general, the enrichment of dairy products with whey-derived peptides is expected to “add” biofunctional ACE-I peptides [36]. Nevertheless, in the present experiments the supplementation of the skim milk base decrease slightly this activity, indicating the importance of the viable counts for this category of dairy products.

## 4. Conclusions

The conditions of the heat treatment of the milk base were more important than the type of enrichment for the physical properties of the gel. These conditions were determined by the heat stability of the mixtures of “versatile” skim sheep milk with WPC or WPHs. The high pressure treatment at conditions that denature almost totally the β-lactoglobulin as an alternative to heating resulted in inferior gel properties, irrespective of the type of enrichment. Supplementation of skim sheep milk with whey protein hydrolysates at > 0.5% had a detrimental effect on gel properties. Finally, the sheep milk base along with the use of fresh traditional yoghurt as a starter, resulted in yoghurt-type gels with high counts of *Lb. delbrueckii* subsp. *bulgaricus* and *Str. thermophilus* -close to the ideal 1:1- and with high ACE-IA, which were not essentially affected by the experimental factors.

## Figures and Tables

**Table 1:** Composition of whey protein concentrate (WP80) g/100 g used as substrate for the production of whey protein hydrolysates (WPHs).

Component	Ca	Mg	K	Na	P	CMP	α-la	SA	β-lg	TNP
g/100 g	0.323	0.065	0.259	0.205	0.214	12.75	14.02	2.63	46.55	75.95

CMP, caseinomacropeptide; α-la, α-lactalbumin, SA, serum albumin; β-lg, β-lactoglobulin; TNP, total native protein.

**Table 2:** Composition and treatments of skim sheep milk bases. Means of two experiments ± standard deviation.

Milk Base	Powder % (*w*/*w*)	Trisodium Citrate	Treatment	pH before Treatment	pH after Treatment
Υ0 ^1^-HT	-	-	95 °C/5 min	6.71 ± 0.09	6.36 ± 0.07
Υ0 ^1^-HP	-	-	600 MPa/55 °C/10 min	6.66 ± 0.01	6.54 ± 0.16
ΥWP80-HT	WP80 ^2^/1%	0.2%	90 °C/5 min	6.78 ± 0	6.52 ± 0.05
ΥWP80-HP		-	600 MPa/55 °C/10 min	6.63 ± 0.02	6.42 ± 0.10
ΥWPHp0.5-HT	WPHp ^3^/0.5%	0.2%	85 °C/5 min	6.85 ± 0.05	6.59 ± 0.03
ΥWPHp0.5-HP		-	600 MPa/55 °C/10 min	6.61 ± 0	6.41 ± 0.07
ΥWPHt0.5-HT	WPHt ^4^/0.5%	0.2%	85 °C/5 min	6.84 ± 0.05	6.55 ± 0.03
ΥWPHt0.5-HP		-	600 MPa/55 °C/10 min	6.60 ± 0.04	6.43 ± 0.11
ΥWPHt1-HT	WPHt ^4^/1%	0.2%	75 °C/5 min	6.80 ± 0.03	6.46 ± 0.09
ΥWPHt1-HP		-	600 MPa/55 °C/10 min	6.61 ± 0.04	6.39 ± 0.10

^1^ defatted sheep milk (control); ^2^ whey protein concentrate powder with approximately 80% (w/w) protein content (WP80) derived from sheep/goat Feta cheese whey; ^3^ trypsin hydrolysate of WP80; ^4^ protamex hydrolysate of WP80.

**Table 3:** Effect of experimental factors on the properties of yoghurt-type gels, expressed as *p*-values.

Properties	A: composition	B: treatment	C: days	A × B	A × C	B × C
pH	0.119	0.634	0	0.034	0.288	0.023
WHC (%)	0	0	0.07	0	0.928	0.371
FIRMNESS (N)	0	0	0.007	0	0.753	0.157
COHESIVENESS	0	0	0.999	0	0.985	0.799
TOTAL SOLIDS (%)	0.001	0.001	-	0.170	-	-
PROTEIN (%)	0.003	0.144	-	0.057	-	-

**Table 4:** Properties of yoghurt-type gels made from skim sheep milk bases enriched with WPHs.

Properties	Treatment	Υ0	ΥWP80	ΥWPHp0.5	ΥWPHt0.5	ΥWPHt1
*Day 3*						
pH	HT ^1^	4.35 ± 0.05 a	4.44 ± 0.02 b	4.33 ± 0.03 a	4.32 ± 0.06 a	4.38 ± 0.04 a, b
	HP ^2^	4.41 ± 0.06	4.35 ± 0.08	4.29 ± 0.06	4.32 ± 0.09	4.32 ± 0.14
WHC (%)	HT	33.21 ± 1.92 b	45.31 ± 1.53 d, B	40,91 ± 2.13 c, B	37.23 ± 1.38 c, B	27.53 ± 2.84 a
	HP	28.52 ± 2.60	29.09 ± 2.21 A	26.72 ± 2.29 A	28.65 ± 1.43 A	28.69 ± 3.52
FIRMNESS (N)	HT	1.40 ± 0.22 b, B	1.78 ± 0.21 b, c, B	1.87 ± 0.24 c, B	1.59 ± 0.14 b, c, B	0.66 ± 0.29 a
	HP	0.70 ± 0.03 c, A	0.35 ± 0.06 a, A	0.52 ± 0.07 b, A	0.69 ± 0.04 c, A	0.57 ± 0.09 b, c
COHESIVENESS	HT	0.426 ± 0.019 a, A	0.421 ± 0.026 a, A	0.422 ± 0.014 a, A	0.449 ± 0.023 a	0.541 ± 0.039 b
	HP	0.540 ± 0.050 a, b, B	0.612 ± 0.019 b, B	0.506 ± 0.029 a, B	0.506 ± 0.021 a	0.546 ± 0.052 a, b
*Day 10*						
pH	HT	4.15 ± 0.07 a *	4.23 ± 0.05 a, b *	4.18 ± 0.03 a *	4.19 ± 0.04 a, b *	4.26 ± 0.02 b *
	HP	4.30 ± 0.04	4.23 ± 0.05	4.22 ± 0.04	4.23 ± 0.02	4.21 ± 0.03
WHC (%)	HT	36.61 ± 1.82 a, b, B	46.49 ± 3.52 c, B	39.74 ± 3.92 b	38.42 ± 3.58 b	30.85 ± 3.96 a
	HP	31.64 ± 1.28 A	29.87 ± 1.83 A	33.85 ± 3.00	31.90 ± 2.14	29.53 ± 2.35
FIRMNESS (N)	HT	1.49 ± 0.36 b	2.20 ± 0.23 c, B	2.04 ± 0.33 c, B	1.83 ± 0.14 b, c, B	0.96 ± 0.15 a
	HP	0.74 ± 0.03 c	0.38 ± 0.02 a, A	0.55 ± 0.03 b, A	0.77 ± 0.01 c, A	0.79 ± 0.02 c
COHESIVENESS	HT	0.429 ± 0.018 a, b	0.408 ± 0.028 a, A	0.438 ± 0.018 a, b	0.456 ± 0.021 b	0.507 ± 0.033 c
	HP	0.527 ± 0.057 a, b	0.646 ± 0.036 b, B	0.454 ± 0.043 a	0.502 ± 0.035 a, b	0.595 ± 0.99 a, b
TOTAL SOLIDS (%)	HT	11.22 ± 0.28 a	12.05 ± 0.30 b	12.10 ± 0.25 b	12.15 ± 0.30 b	12.53 ± 0.21 c, B
	HP	11.25 ± 0.21	11.98 ± 0.25	11.80 ± 0.35	11.58 ± 0.32	11.45 ± 0.21 A
PROTEIN (%)	HT	4.73 ± 0.18 a	5.28 ± 0.23 b, c	5.15 ± 0.17 b	5.13 ± 0.24 b	5.59 ± 0.20 c
	HP	4.80 ± 0.14 a	5.40 ± 0.14 b	4.93 ± 0.18 a	5.18 ± 0.18 a, b	4.98 ± 0.25 a, b

Means of two experiments ± standard deviation. Symbols as in Table 2; ^1^ heat treatment conditions indicated in Table 2; ^2^ high hydrostatic pressure treatment at 600 MPa/55 °C/10 min. a–d lowercase letters indicate statistically significant differences (LSD, *p* < 0.05) within each type of treatment, i.e., within rows; A–B, indicate significant differences (*p* < 0.05) between heat treatments (HT) and high pressure (HP) treatments; * indicates significant differences (*p* < 0.05) between three and 10 days.

**Table 5:** Biofunctional characteristics of yoghurt-type gels made from skim sheep milk bases enriched with WPHs after ten days of storage.

Parameter	Treatment	Υ0	ΥWP80	ΥWPHp0.5	ΥWPHt0.5	ΥWPHt1	Factors/*p*-Values ^5^		
							A: composition	B: treatment	A × B
*Day 10*									
*Lb. bulgaricus*^3^ (log cfu/g)	HT ^1^	8.20 ± 0.38	8.08 ± 0.25	8.26 ± 0.12 B	8.40 ± 0.12	8.23 ± 0.21	0.294	0	0.482
	HP ^2^	7.39 ± 0.34	7.83 ± 0.21	7.64 ± 0.32 A	7.92 ± 0.28	7.91 ± 0.33			
*Str. thermophilus*^4^ (log cfu/g)	HT	6.74 ± 0.41	7.28 ± 0.43	7.36 ± 0.33	7.52 ± 0.61	7.36 ± 0.81	0.327	0.965	0.564
	HP	6.82 ± 0.71	7.91 ± 0.77	7.38 ± 0.45	6.79 ± 0.86	7.43 ± 0.67			
ACE-IA (%)	HT	76.40 ± 1.95 b	73.97 ± 3.23 a, b	73.43 ± 2.04 a, b	70.9 ± 1.15 a B	73.53 ± 3.52 a, b	0.019	0.009	0.542
	HP	73.25 ± 4.60	74.2 ± 4.95	69.3 ± 2.97	64.65 ± 3.32 A	68.15 ± 2.90			

Means of two experiments ± standard deviation. Symbols as in Table 2; ^1^ heat treatment conditions indicated in Table 2; ^2^ high hydrostatic pressure treatment at 600 MPa/55 °C/10 min; ^3^ *Lactobacillus delbrueckii* subsp. *bulgaricus* [34]; ^4^ *Streptococcus thermophilus* [34]; ^5^ two-way ANOVA. a–b, lowercase letters indicate statistically significant differences (LSD, *p* < 0.05) within each type of treatment, i.e., within rows; A–B, indicate significant differences (*p* < 0.05) between HT and HP treatments.

## Abbreviations

ACE-IA	angiotensin-converting enzyme- inhibitory activity
CMP	caseinomaropeptide
cfu	colony forming unit
RP-HPLC	reversed-phase, high performance liquid chromatography
SA	serum albumin
TNP	total native protein
α-la	α-lactalbumin
β-lg	β-lactoglobulin
